# Supplementary material for: Associations of Vitamin D Deficiency, Parathyroid hormone, Calcium, and Phosphorus with Perinatal Adverse Outcomes. A Prospective Cohort Study
Source: Nutrients. 2020 Oct 26;12(11):3279. doi: 10.3390/nu12113279 (PMC7692385; doi:10.3390/nu12113279)
Supplement: Supplementary file 1 [file nutrients-12-03279-s001.pdf]

Table S1. Associations between combination of maternal serum 25-hydroxyvitamin D and PTH 75th percentile and perinatal adverse outcomes.

	Preterm birth			Low birth weight			Small for gestational age		
	**n (%)**	**OR**	**aOR[1]**	**n (%)**	**OR**	**aOR[2]**	**n (%)**	**OR**	**aOR[3]**
**25[OH]D ≥20 ng/mL (≥50 nmol/L)**									
**PTH >75th**	0/35 (0%)	--	--	0/35 (0%)	--	--	0/35 (0%)	--	--
**PTH ≤75th**	7/149 (4.7%)	0.651 (0.237 – 1.733)	0.649 (0.226 – 1.865)	11/149 (7.4%)	0.779 (0.337 – 1.801)	1.100 (0.373 – 2.733)	13/149 (8.7%)	0.860 (0.389 – 1.901)	0.957 (0.429 – 2.218)
**25[OH]D <20 ng/mL (<50 nmol/L)**									
**PTH >75th**	7/37 (18.9%)	5.647 (2.000 – 15.939) *	6.292 (2.054 – 19.271) *	7/37 (18.9%)	3.225 (1.237 – 8.413) *	1,562 (0.446 – 5.469)	6/37 (16.2%)	2.129 (0.798 – 5.682)	1.706 (0.596 – 4.885)
**PTH ≤75th**	3/68 (4.4%)	0.682 (0.190 – 2.449)	0.816 (0.210 – 3.170)	6/68 (8.8%)	1.091 (0.415 – 2.870)	1.305 (0.411 – 4.142)	8/68 (11.8%)	1.418 (0.591 – 3.400)	1.418 (0.575 – 3.498)
**25[OH]D ≥30 ng/mL (≥75 nmol/L)**									
**PTH >75th**	0/4 (0%)	--	--	0/4 (0%)	--	--	0/4 (0%)	--	--
**PTH ≤75th**	2/30 (6.7%)	1.162 (0.253 – 5.346)	1.480 (0.310 – 7.065)	1/30 (3.3%)	0.354 (0.460 – 2.718)	0.257 (0.024 – 2.787)	1/30 (3.3%)	0.309 (0.040 – 2.363)	0.324 (0.041 – 2.548)
**25[OH]D <30 ng/mL (<75 nmol/L)**									
**PTH >75th**	7/68 (10.3%)	2.421 (0.885 – 6.628)	2.060 (0.692 – 6.132)	7/68 (10.3%)	1.377 (0.546 – 3.474)	0.808 (0.252 – 2.585)	6/68 (8.8%)	0.922 (0.356 – 2.385)	0.812 (0.303 – 2.181)
**PTH ≤75th**	8/187 (4.3%)	0.462 (0.173 – 1.236)	0.478 (0.166 – 1.374)	16/187 (8.6%)	1.099 (0.454 – 2.664)	1.908 (0.607 – 6.600)	20/187 (10.7%)	1.625 (0.663 – 3.985)	1.877 (0.735 – 4.798)

Data reported as OR (95%CI). [1] Adjusted for pre-eclampsia and history of preterm birth. [2] Adjusted for maternal age, smoking habit, pre-eclampsia, and preterm birth. [3] Adjusted for seasonality, smoking habit, and parity. * p-value < 0.05.
